# Experimental Characterization and Mathematical Modeling of the Adsorption of Proteins and Cells on Biomimetic Hydroxyapatite

**DOI:** 10.1021/acsomega.1c05540

**Published:** 2021-12-22

**Authors:** Abdul-Raouf Atif, Uǵis La̅cis, Håkan Engqvist, Maria Tenje, Shervin Bagheri, Gemma Mestres

**Affiliations:** † Department of Materials Science and Engineering, Uppsala University, Box 35, 751 22 Uppsala, Sweden; ‡ Department of Engineering Mechanics, FLOW Centre, KTH Royal Institute of Technology, 114 28 Stockholm, Sweden; § Science for Life Laboratory, Uppsala University, 751 22 Uppsala, Sweden

## Abstract

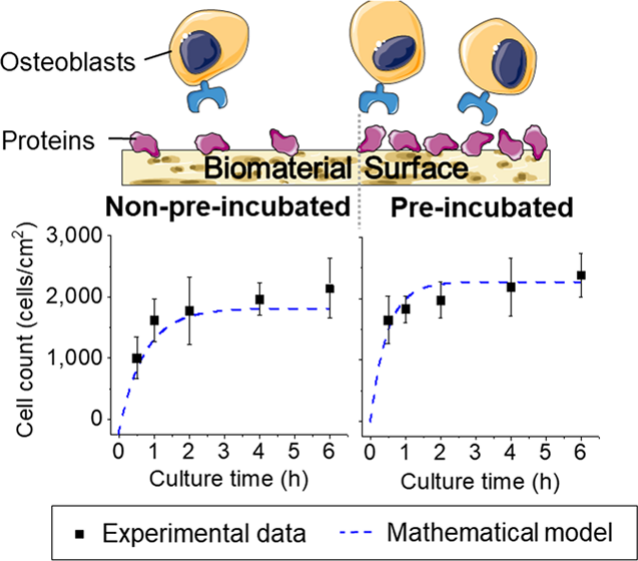

Biomaterial development is a long process consisting of multiple stages of design and evaluation within the context of both *in vitro* and *in vivo* testing. To streamline this process, mathematical and computational modeling displays potential as a tool for rapid biomaterial characterization, enabling the prediction of optimal physicochemical parameters. In this work, a Langmuir isotherm-based model was used to describe protein and cell adhesion on a biomimetic hydroxyapatite surface, both independently and in a one-way coupled system. The results indicated that increased protein surface coverage leads to improved cell adhesion and spread, with maximal protein coverage occurring within 48 h. In addition, the Langmuir model displayed a good fit with the experimental data. Overall, computational modeling is an exciting avenue that may lead to savings in terms of time and cost during the biomaterial development process.

## 1 Introduction

Careful evaluation of novel biomaterials is necessary to ensure complete fulfillment of their intended function. The assessment includes characterization of physicochemical and biocompatibility properties, with the latter implying subsequent *in vitro* and *in vivo* studies.^1^ However, the lack of correlation between *in vitro* and *in vivo* assays^2^ leads to an iterative, long, and expensive process.^3^ This could partially explain why, despite extensive biomaterial developments over the last decades, only a small fraction of biomaterials have been fully translated into the clinical environment.^4,5^

While computational modeling has been revolutionary in discovering new therapeutics, its usage has not yet been fully extended into the development of new biomaterials, despite its promising potential.^6^ Within the field of biomaterial development, computational models have been used to predict the macroscopic mechanical behavior of materials,^7^ mainly using either finite-element methods or simple mathematical expressions that define the parameter under study.^8,9^ Computational modeling is also useful in predicting experimental results during the biological characterization of biomaterials. For example, Chen et al. developed a cell adhesion model to identify the ideal alumina grain size to enhance osteoblast adhesion.^10^ Likewise, Sanz-Herrera et al. developed a multiscale model to demonstrate that scaffold stiffness and pore size directly correlate with the regeneration rate of bone.^11^ The degradation of magnesium-based implants both *in vitro* and *in vivo*,^12^ as well as the bone regeneration and turnover process have also been studied with the use of computational models.^13,14^

Overall, the combination of experimentation with computer modeling allows for the potential interrelation of several physicochemical properties of biomaterials (e.g., roughness, surface charge, or porosity) and how they may influence the interaction with biological moieties and cell function.^15,16^ Such approaches may constitute time- and cost-effective strategies to provide predictive information and guide the tuning of biomaterials’ physicochemical properties prior to the beginning of *in vivo* studies.

When a biomaterial is implanted in the body, it comes in contact with biological fluids (e.g., blood), thus enabling rapid protein adsorption on its surface.^17^ Among the variety of blood plasma proteins, albumin is the most abundant,^18^ followed by other proteins such immunoglobin, fibrinogen, or fibronectin.^17^ While smaller proteins tend to interact first with the surface and adsorb onto it, larger proteins may eventually displace them in a process known as the Vroman effect.^19^ The plasma proteins adsorbed on the surface can interface and selectively bind with transmembrane receptors of cells such as integrins, thus anchoring the cell on the substrate. In other words, the cells do not directly interact with the “naked” biomaterial surface, but rather with a dynamic coating of proteins.^17^ Better understanding of the protein-biomaterial interplay will contribute to improving the biocompatibility of new implants.

In this work, a modular numerical model was formulated to describe the coupling between protein adhesion and subsequent osteoblast attachment on biomimetic hydroxyapatite (HA). For this purpose, the adsorption and desorption of albumin on HA were monitored, as well as the adhesion of osteoblasts on HA previously incubated with serum-conditioned medium for different times. Using the experimental data, two modules were developed using the Langmuir isotherm for both protein and cell adhesion to HA. The connection between the two modules and the coupling model between protein and cell interaction is discussed. This work provides the first building blocks toward using multi-physics computational modeling for the evaluation of cell interactions on biomaterial surfaces.

## 2 Results

### 2.1 Characterization of Biomimetic HA

**Figure 1:**
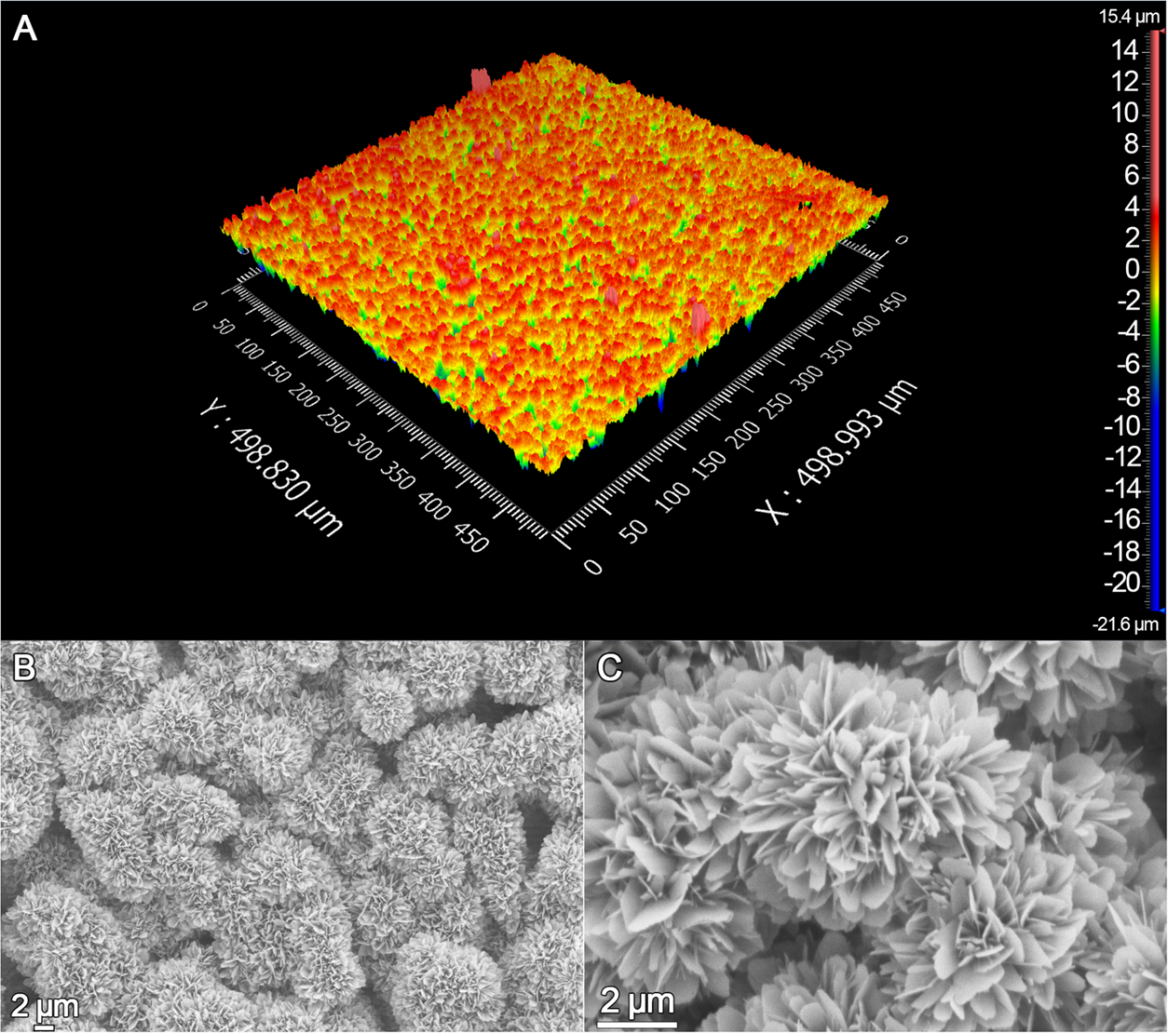
Characterization of the biomimetic HA surface. (A) Optical profilometry of a 500 × 500 μm^2^ area of the HA surface, with surface variations indicated quantitatively using a color scale, with a red gradient indicating the highest areas and a green and blue gradient indicating the lowest areas on the surface. (B, C) Representative SEM images of the material surface showing characteristic HA crystals taken at two different magnifications.

Surface analysis of biomimetic HA was performed using both optical profilometry and scanning electron microscopy (SEM). Profilometry analysis indicated that higher-order variations in surface topography of HA homogenously varied between 4 and −4 μm, and the measured average roughness of the surface (*S*_a_) was 1.4 μm (Figure 1A). SEM imaging showed the presence of entangled crystals on the surface of the material, specifically surrounding the original granules of α-TCP (Figure 1B). The plate-like crystal morphology observed is characteristic of apatite cement with α-TCP particles within a range of 0.5–100 μm, which set through a dissolution–precipitation mechanism at low temperature (Figure 1C). The assessment of crystalline phases by X-ray diffraction confirmed a quantitative transformation of the initial α-TCP into HA (Figure S.I. 1).

### 2.2 Experimental Adsorption and Desorption of Protein on Biomimetic HA

**Figure 2:**
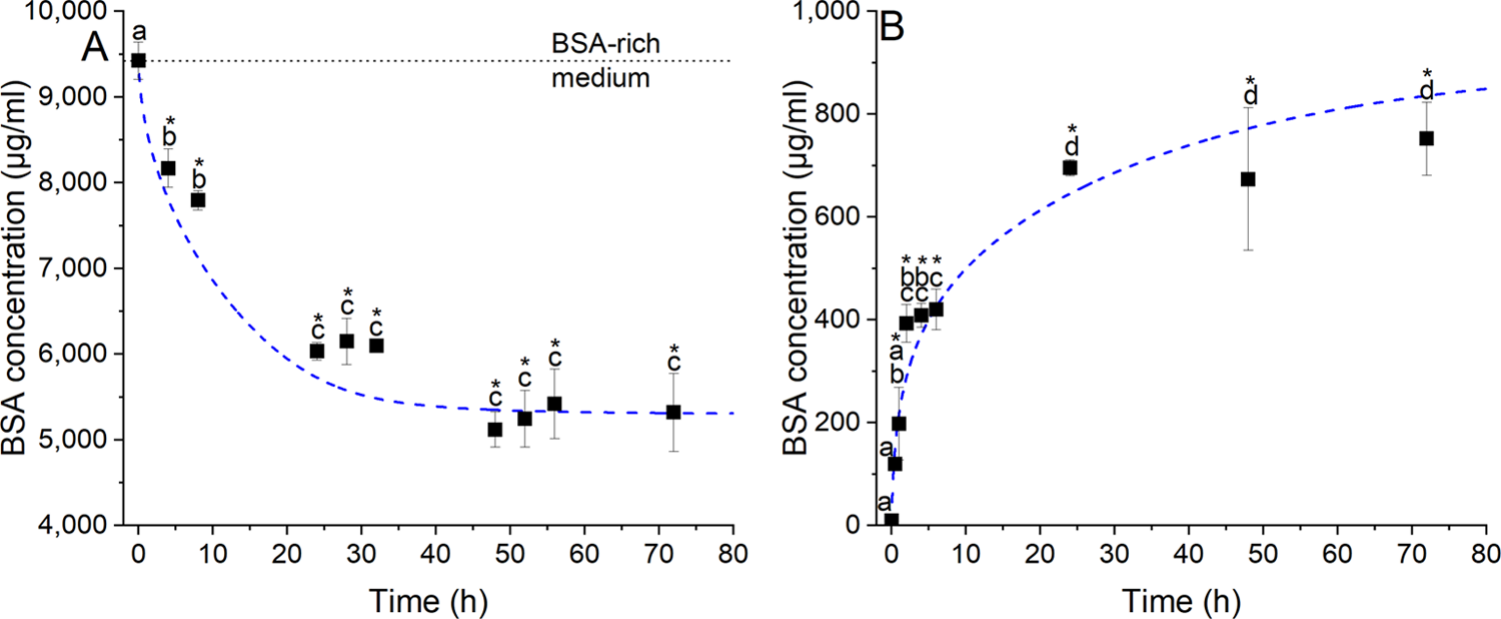
BSA concentration in solution for (A) adsorption on and (B) desorption from the biomimetic HA surface against time. The individual points indicate the experimental data, and the dashed trace corresponds to the model prediction (eqs 1–3); * indicates statistical significance between data points and starting medium conditions, specifically against BSA-rich and BSA-free medium for adsorption and desorption, respectively (*p* < 0.05). Identical letters indicate no statistical significance between time points within each experiment (*p* < 0.05).

The adsorption and subsequent desorption of BSA on biomimetic HA were investigated through incubation of HA in BSA-rich and BSA-free medium, respectively. HA adsorbed almost half of the total BSA content (45 ± 3.6%, *p* < 0.001) in the medium within the first 48 h. Afterward, the stable levels of BSA in the medium indicated that an equilibrium had been reached between the surface and BSA fluid phase (Figure 2A). Subtracting the final (5300 μg/mL) from the initial (9400 μg/mL) concentration of BSA in the fluid, the final concentration entering the surface was calculated to be 4100 ± 670 μg/mL, which corresponded to a surface concentration of 29.04 μg/mm^2^.

After evaluation of the adsorption profile, the HA samples were transferred into BSA-free DMEM to evaluate the desorption of BSA over time. BSA was initially released rapidly from the HA surface (∼3.2 ± 0.37 μg/mL·min for the first 2 h), but subsequently experienced a decline in release (∼0.23 ± 0.04 μg/mL·min) before reaching a plateau at 24 h, indicating the establishment of an equilibrium state of BSA concentration between the HA surface and the fluid phase. The total amount of BSA released was 700 ± 16 μg/mL (Figure 2B), which represented ∼18 ± 1.2% of the BSA previously attached.

#### 2.2.1 Modeling of Protein Adsorption and Desorption

Using the experimental data, the final steady-state fluid phase protein concentration (*c*^f^) and the final surface protein concentration (*c*_s_^f^) were determined to be 5300 μg/mL and 29.04 μg/mm^2^, respectively. Since the adsorption and desorption coefficients (*k*_a_ and *k*_d_, respectively) cannot be estimated experimentally, they were treated as free parameters, as explained in Section 5.5. The adsorption and desorption coefficient values were determined to be *k*_a_ = 6.2 × 10^–4^ mL·μg^–1^·h^–1^ and *k*_d_ = 0.2 h^–1^. The corresponding maximum protein surface concentration was *c*_s_^m^ = 31.5 μg/mm^2^. These values provided the smallest error between simulated and experimental results, computed using eq 5. The model prediction of protein concentration in the solution over time is shown in Figure 2A,B along with the experimental data. As can be observed, the model follows the general trend of the experimental results and fit within the experimental error bars at most time points, thus indicating a good degree of agreement.

**Figure 3:**
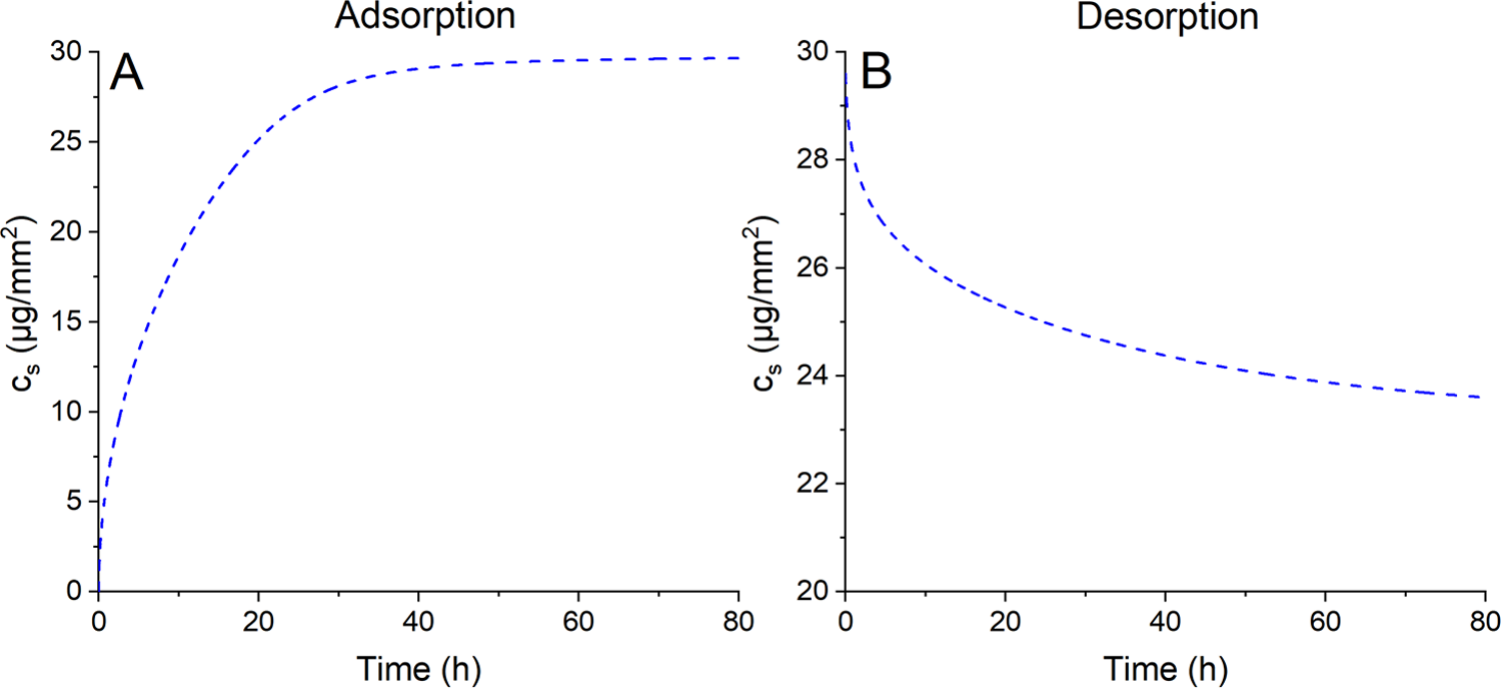
Mathematical model for protein concentration on the HA surface (eq 3) during the (A) adsorption and (B) desorption process on the HA surface.

While the concentration of BSA protein on the substrate surface was not assessed experimentally, the prediction obtained via modeling is displayed in Figure 3. The results indicate that, during the adsorption process, the steady saturation state was reached after around 40 h (Figure 3A). In contrast, while initially rapid, a slower release of protein was observed during the desorption phase (Figure 3B), which did not reach yet a fully steady concentration by 80 h. The modeling data indicated that 20.6% of adsorbed protein was released back into the solution after 80 h.

### 2.3 Cell Adhesion on Biomimetic HA

The number of cells adsorbed on HA and TCPS was evaluated. To correlate cell adhesion to protein adsorption, different substrates were pre-incubated in supplemented medium for 24 or 48 h, or were directly used without any pre-incubation step (0 h) prior to cell seeding.

**Figure 4:**
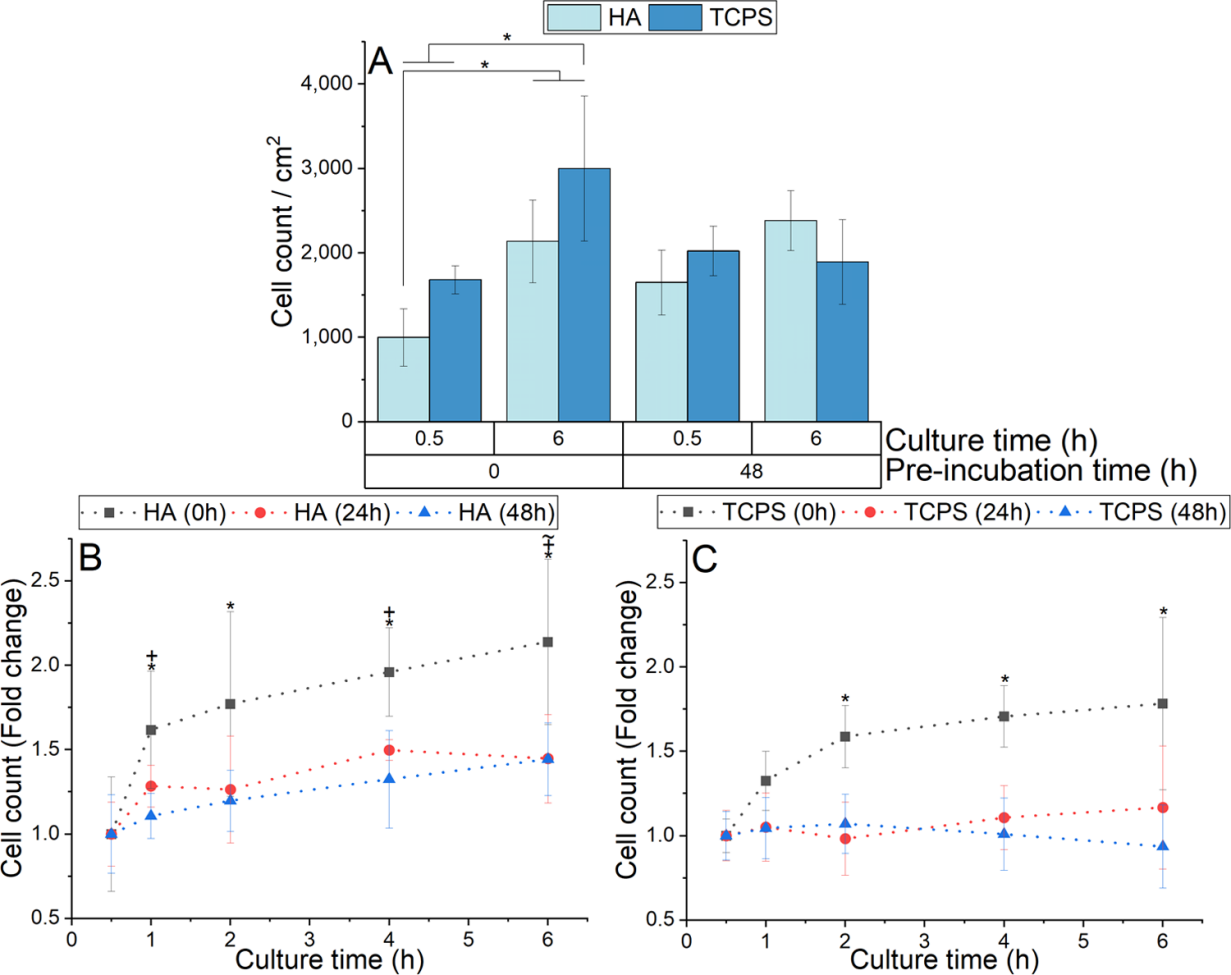
(A) Comparison of HA and TCPS cell counts at 0.5 and 6 h of culture for pre-incubation times of 0 and 48 h; * indicates statistical significance between specified samples (*p* < 0.05). (B) HA and (C) TCPS were used without pre-incubation (0 h) or pre-incubated for 24 and 48 h, and adhered cells were counted at 0.5, 1, 2, 4, and 6 h of culture; *, +, and ∼ indicate statistical significance between marked samples and the respective sample taken at 0.5 h for 0, 24, and 48 h conditions, respectively. Note that the results are expressed as cell counts in (A) and, in (B) and (C) as fold-change relative to the sample taken at 0.5 h of culture time.

Regarding the extent of cell adhesion on HA samples, a general increase in cell count over time was observed for the three pre-incubation times investigated (Figure 4B). When comparing the cell counts on non-pre-incubated samples (0 h), a larger number was present on TCPS in comparison to HA samples, both at 0.5 and 6 h of culture (Figure 4A). However, the difference between sample types was reduced after pre-incubation for 48 h, at both culturing times (Figure 4A). Over the entire cell culture, non-pre-incubated HA samples (0 h) showed the largest increase in cell count (*p* < 0.001 between 0.5 and 6 h time points), while HA samples pre-incubated for 24 and 48 h displayed a slight increase over time (*p* < 0.001 and *p* = 0.018, respectively, comparing 0.5 and 6 h time points) (Figure 4B). Similarly, non-pre-incubated TCPS samples showed the greatest increase in cell count over time, specifically during the first 2 h of cell culture (*p* = 0.006 between 0.5 and 2 h time points) (Figure 4C). On the other hand, TCPS samples pre-incubated for 24 and 48 h showed virtually no increase in cell count for the entire study (Figure 4C).

**Figure 5:**
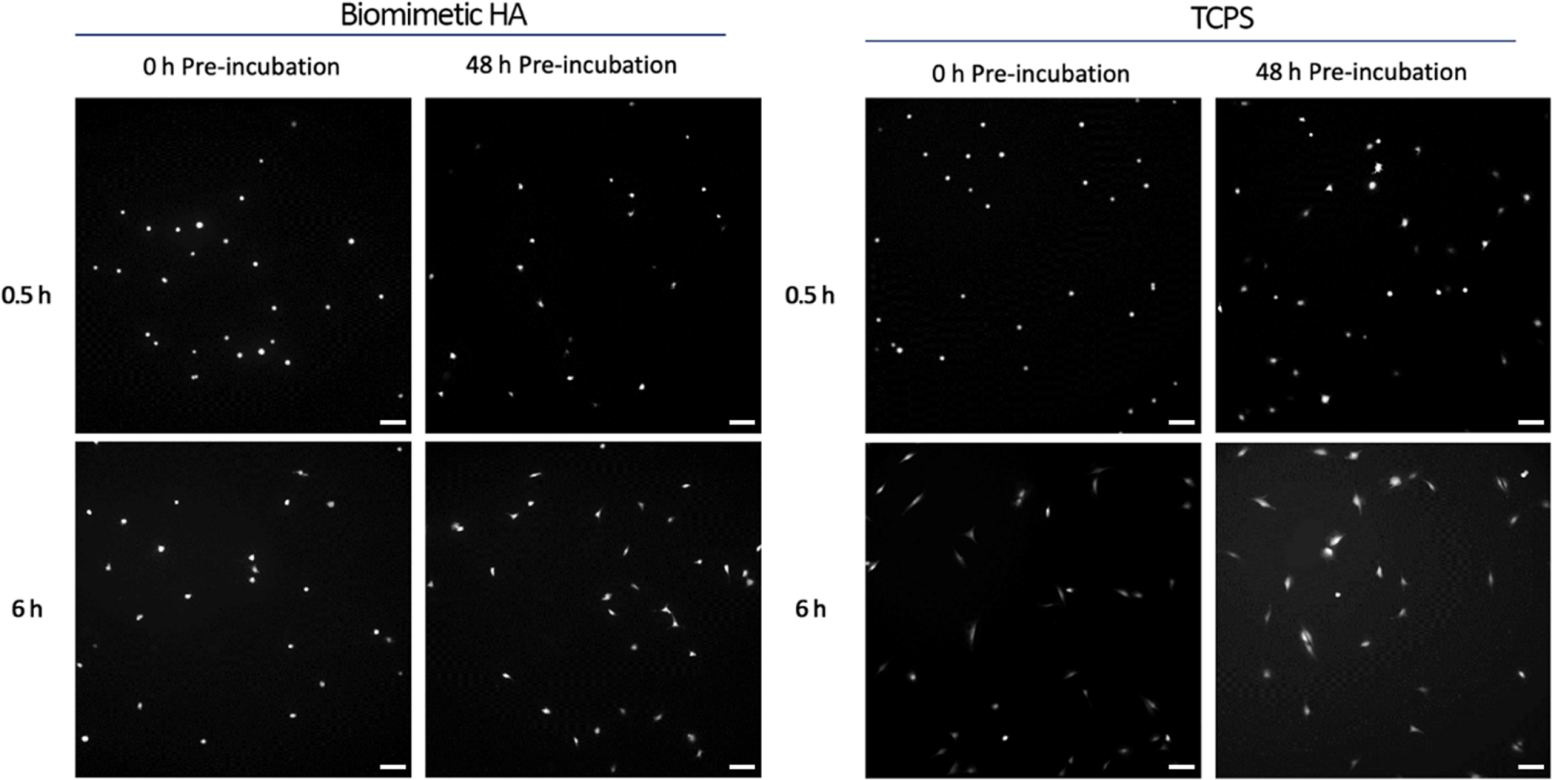
Cell adhesion on HA and TCPS after 0.5 and 6 h of culture, with each sample pre-incubated for 0 or 48 h (scale bar = 100 μm).

The morphology of the cells seeded on HA and TCPS, which were pre-incubated for different time periods (0, 24, and 48 h), was monitored over a period of 6 h (Figures 5, S.I. 2, and S.I. 3). Interestingly, while cells showed a round morphology when seeded on non-pre-incubated HA, they were able to spread after 6 h of culture, this effect being amplified if the HA had been pre-incubated. In the case of TCPS, cells showed a clear degree of cell spread after 6 h of culture regardless of the pre-incubation duration.

**Figure 6:**
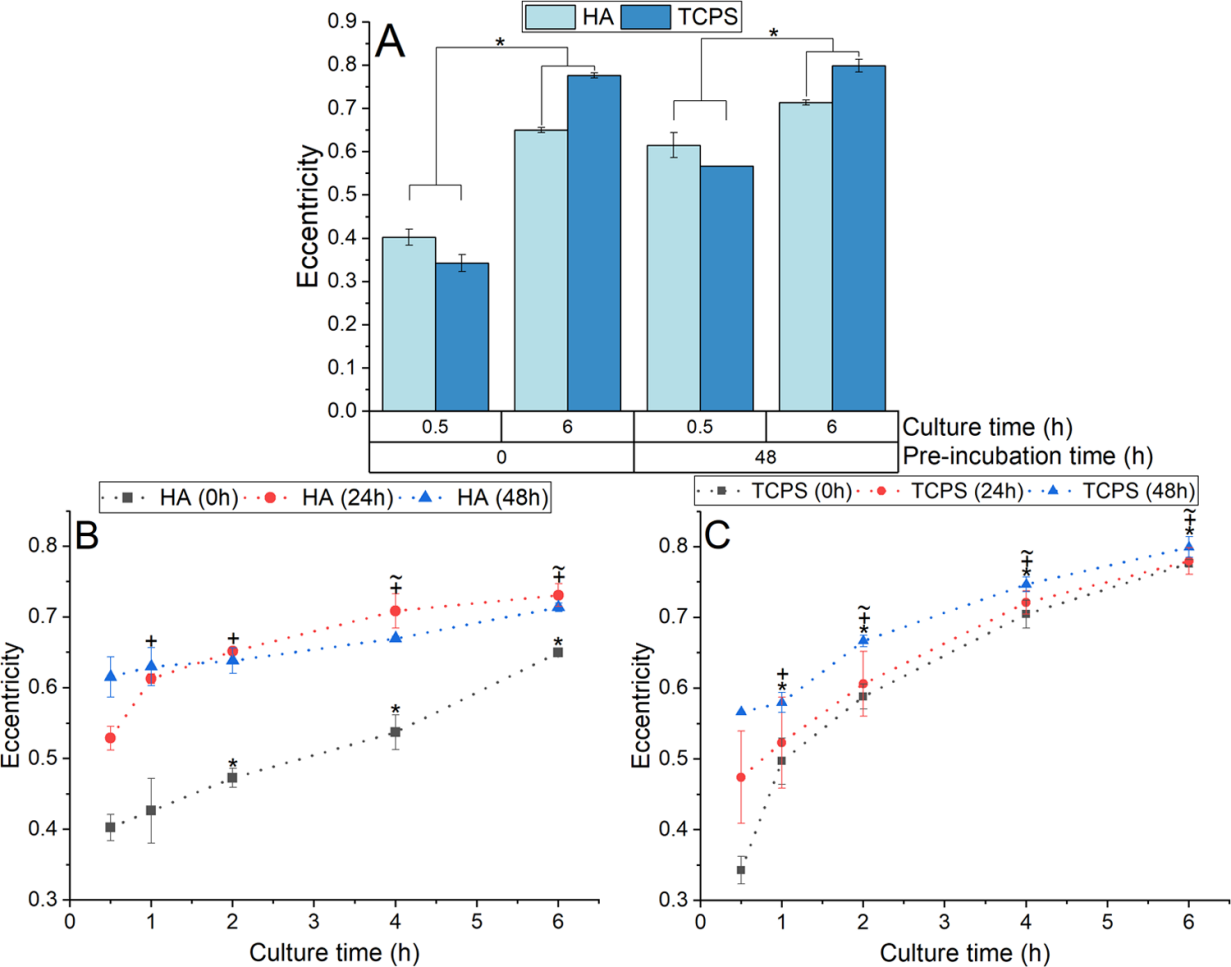
(A) Comparison of eccentricity between HA and TCPS samples pre-incubated for 0 or 48 h and subsequently cultured for 0.5 and 6 h; * indicates statistical differences between specified samples (*p* < 0.05). (B) Eccentricity of cells adhered on HA and (C) TCPS at 0.5, 1, 2, 4, and 6 h of culture with samples pre-incubated for 0, 24, and 48 h; *, +, and ∼ indicate the statistical difference between the indicated sample and the respective samples cultured for 0.5 h and pre-incubated for 0, 24, and 48 h, respectively.

The extent of morphological spread of adhered cells on both surfaces (Figure 5) was quantified as eccentricity, where a value close to 1 indicates an elongated cell morphology. When comparing cellular eccentricity values between HA and TCPS, a clear trend was observed. For pre-incubation periods of 0 and 48 h, while eccentricity was higher in HA than TCPS samples at a culture period of 0.5 h (comparison for both 0 and 48 h pre-incubations, *p* < 0.001 and *p* = 0.001, respectively), interestingly, the reverse situation was observed at 6 h of culture time (comparison between HA and TCPS for both 0 and 48 h pre-incubations, *p* < 0.001) (Figure 6A). The eccentricity of cells on HA samples was significantly higher for samples pre-incubated for 24 and 48 h compared to non-pre-incubated samples (at 6 h of culture, *p* < 0.001) (Figure 6B). For both HA and TCPS samples, eccentricity increased over time for all three pre-incubation times, with this increase being more pronounced for non-pre-incubated samples (Figure 6B,C). Specifically for TCPS, at each incubation time, the eccentricity values for 48 h pre-incubated samples were consistently higher than for 0 h pre-incubated samples (up to 4 h of culture, *p* < 0.001) (Figure 6C). The most notable distinction in eccentricity between samples was observed at 0.5 h, where cells cultured on TCPS samples pre-incubated for 0, 24 and 48 h showed values of 0.34 ± 0.02, 0.47 ± 0.07, and 0.57 ± 0.0002, respectively.

#### 2.3.1 Modeling of Cell Adhesion on Biomimetic HA

**Figure 7:**
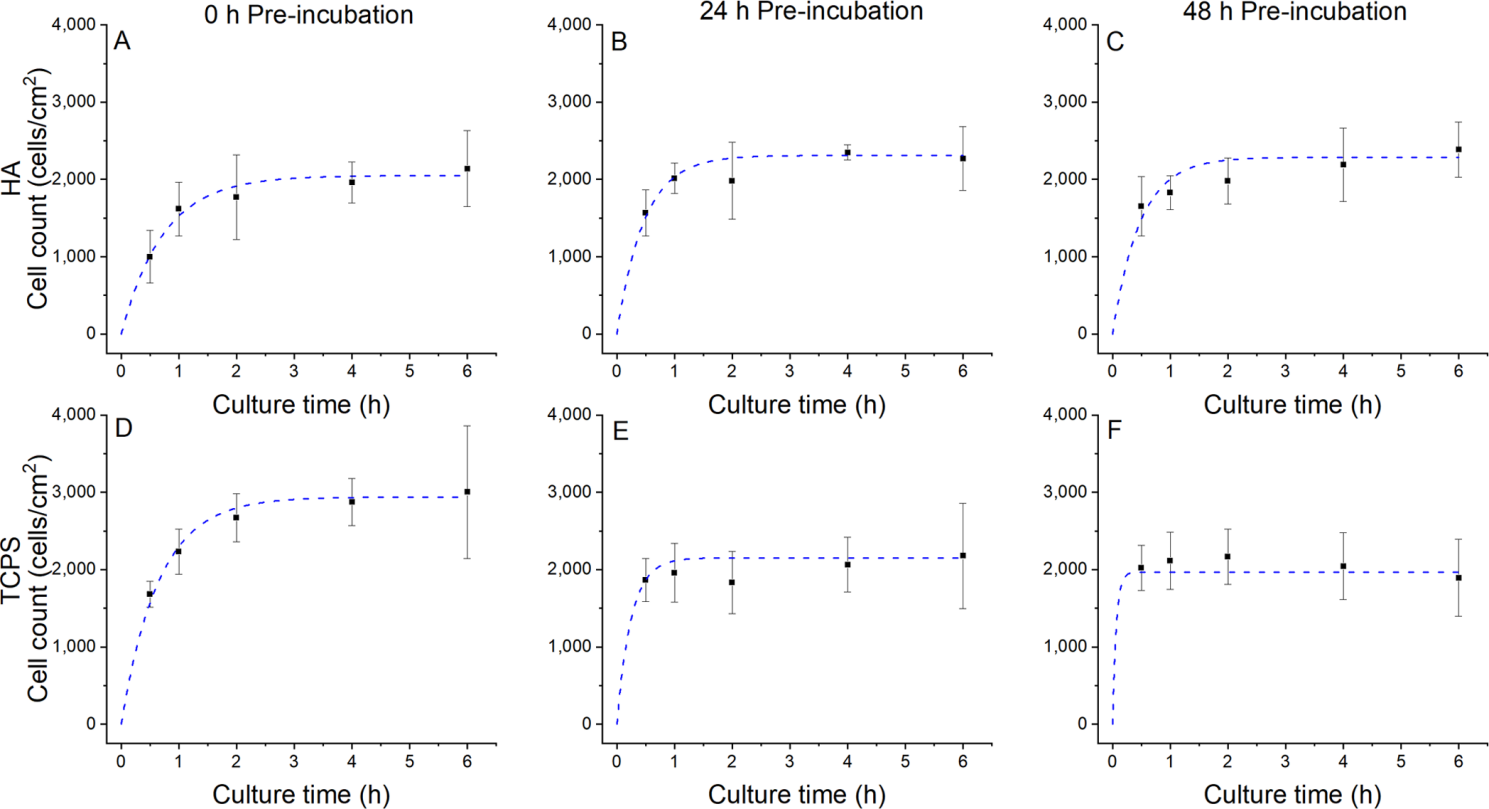
Mathematical modeling for cell adhesion as a function of culture time for HA pre-incubated for (A) 0 h, (B) 24 h, and (C) 48 h and TCPS pre-incubated for (D) 0 h, (E) 24 h, and (F) 48 h. Adhesion constant *k*_a_^OB^ is fitted individually for each set of experimental data. The dashed trace corresponds to the analytical model (obtained with eq 9) with *k*_a_^OB^ constant in time. The points displaying error bars correspond to experimental data.

The cell adhesion coefficient *k*_a_^OB^ was fitted to match the experimental cell adhesion results described in Section 2.3. The best-fit model results, along with experimental results are shown in Figure 7. A good agreement between the model and the experimental data was found, with the model line always being positioned within the interval indicated by error bars. The obtained survival fraction, γ, adhesion constant, *k*_a_^OB^, and final modeling error for all experimental data are shown in Table 1.

**Table 1:** Model Parameters Obtained for Cell Adhesion Experimental Results (Survival Fraction γ and Adhesion Constant *k*_a_^OB^) and Modeling Error with Respect to the Center Points of Experimental Measurements^a^

	HA			TCPS		
pre-incubation time [h]	0	24	48	0	24	48
survival fraction γ	0.59 ± 0.10	0.66 ± 0.07	0.65 ± 0.07	0.84 ± 0.17	0.62 ± 0.16	0.56 ±0. 17
adhesion constant *k*_a_^OB^ [×10^–5^ cm^2^/cells·h]	0.86 ± 0.17	1.33 ± 0.37	1.31 ± 0.47	0.96 ± 0.14	2.53 ± 0.86	6.42 ± 3.77
modeling error [%]	5.36	6.63	4.46	3.52	5.69	5.11
surface protein concentration *c*_s_ [μg/mm^2^]	9.78	27.5	29.4			

a Expected surface protein concentration for biomimetic HA is also reported (experiments not performed with TCPS).

**Figure 8:**
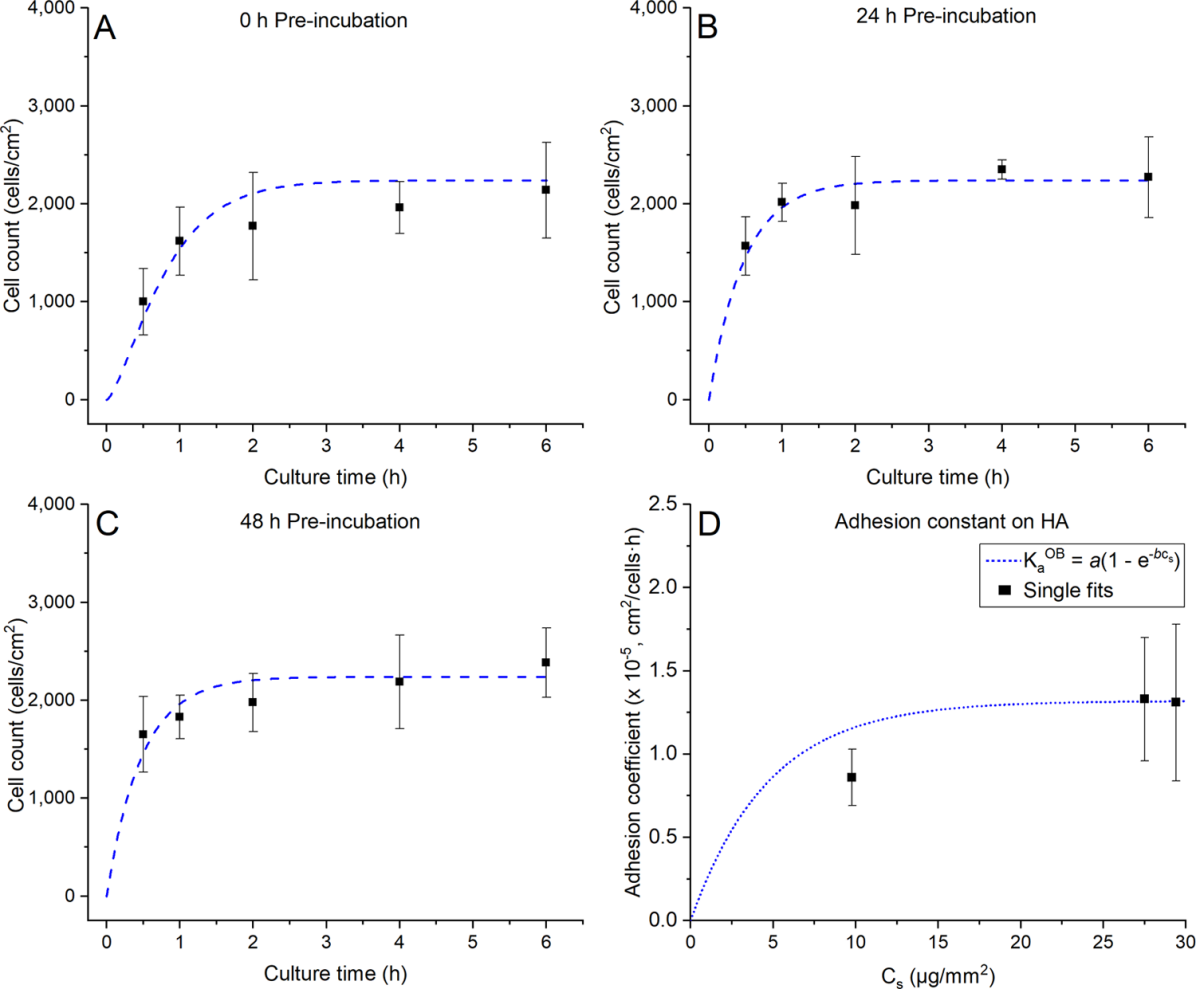
Mathematical model coupling protein adhesion to cell adhesion on HA (eqs 9 and 10) as a function of culture time at pre-incubation times of (A) 0 h, (B) 24 h, and (C) 48 h. The dashed trace corresponds to the coupled cell adhesion model, and the points correspond to experimental data. (D) Selected transfer function to identify the cell adhesion coefficient based on surface protein concentration.

In the set of results described to this point, the modeling of cell adhesion was carried out independently from protein adsorption/desorption. To couple the two processes, the correlation between cell model parameters (γ, *k*_a_^OB^) and protein model results (*c*_s_) was sought. To obtain the surface protein concentration (*c*_*s*_) value for each HA pre-incubation time, an average value of the protein surface concentration was computed for each time span (pre-incubation time in addition to 6 h of cell culture experiment). Figure S.I. 4 graphically shows the resulting *c*_*s*_ values for each pre-incubation time, which are also numerically summarized in Table 1. Since the cell survival fraction (γ) did not change with respect to different surface protein concentrations within the error bounds, a constant mean value from all experiments was used (γ = 0.63 ± 0.08). In contrast, the cell adhesion constant *k*_a_^OB^ exhibited a dependence on surface protein concentration, *c*_s_. To obtain a suitable transfer function, the values for *a* and *b* in eq 10 were altered (using the values presented in Table 1 as guidelines) while comparing the experimental results with the cell adhesion model. A good agreement between the coupled cell adhesion model and the experiments was obtained for *a* = 1.32 × 10^–5^ cm^2^·cells^–1^·h^–1^ and *b* = 0.213 mm^2^·μg^–1^ (Figure 8). The *k*_a_^OB^ (c_s) along with the individual constant *k*_a_^OB^ values are presented in Figure 8D.

## 3 Discussion

To develop a successful computational model for cell adhesion on a biomaterial surface, three key requirements should be fulfilled to ensure both reliability and reproducibility. First, the model must contain the key factors involved in the cell adhesion process, such as protein adsorption/desorption on the surface and the subsequent cell adhesion onto this protein layer. Second, the model should be able to provide versatility to modify individual parameters. Third, while the ability to describe the overall thermodynamic process (protein or cell adherence) cannot be compromised, each part of the model should be as simple as possible and allow an understandable interaction between different model components.

The adsorption of a model protein on HA was assessed through incubation of HA samples in a BSA-rich medium. Under physiological pH, BSA is negatively charged (isoelectric point = 4.8),^20^ thus it is expected to bond on the HA’s C-sites.^21^ The adsorption of BSA on the substrate was observed as a decrease of BSA in the medium at a rate of ∼141.3 ± 13.3 μg/mL·h over the first 24 h and 38.1 ± 12.8 μg/mL·h over the next 24 h. After 48 h, no uptake of BSA from the medium was detected, indicating that equilibrium had been reached. A rate of BSA uptake (119 μg/mL·h) was previously reported by Espanol et al. who observed BSA content (initial concentration of 7000 μg/mL) drop by more than half after 40 h in contact with HA disks (L/P ratio = 0.65),^22^ which was similar to what was observed in the current study (115 μg/mL·h for the same time period).

The BSA desorption study revealed that BSA was rapidly released in a “burst-like” manner from the HA surface (192 ± 24 μg/mL·h) within the first 2 h of incubation, a noticeably more rapid rate compared to BSA adsorption. Interestingly, BSA release decreased by 24 h with 82 ± 1.2% of BSA still remaining in the HA. The low amount of BSA released could be largely attributed to the fact that HA is a highly microporous material and a large portion of BSA molecules would penetrate into the material, reaching a depth of around 250 μm according to Espanol et al.^22^ Another study also demonstrated rapid protein desorption from BSA-loaded HA nanoparticles during the first 48 h of incubation, followed by a slower release, with a 70% of total BSA content being released after 240 h of incubation.^23^ In that case, the larger amount of BSA released in comparison to the current study could be associated with the higher specific surface area of the nanoparticles compared to bulk HA disks, therefore enabling a more efficient desorption process.

BSA adsorption/desorption on HA was successfully modeled with a good fit to the experimental data, where modeling errors were below 6% for adsorption experiments and below 12% for desorption experiments. This demonstrates that a simple model based on the Langmuir isotherm is sufficient to model both adsorption and desorption processes of proteins to a geometrically complex substrate.^24,25^ A weakness of the model is assumption A3, where the biomaterial was considered infinitely thin, while, in fact, proteins can penetrate through the bulk of the microporous material.^22^ However, the good agreement between the model and experiments (Figure 2) indicates that the bulk penetration is not the main effect in the currently studied process. The ability of the Langmuir isotherm to define the equilibrium created between BSA and lysozyme protein to an HA surface was previously investigated by Kandori et al. (protein concentrations ranging from 0 to 19 mg/cm^3^ and pH conditions from 4 to 12) and Lee et al. (HA treated with different amino acids).^26,27^ The latter study demonstrated that the equilibrium was slightly better described with a Freundlich isotherm than with the Langmuir isotherm.^27^ We however demonstrated that the Langmuir isotherm can describe the time evolution of protein concentration sufficiently well.

Cell adhesion was studied with MC3T3-E1 pre-osteoblasts cultured on HA and TCPS surfaces. The samples were previously incubated in a medium supplemented with 10% FBS for set time periods to couple the influence of cell adhesion to the amount of protein on the surface. The number of cells adhered on non-pre-incubated HA significantly increased from 0.5 to 6 h, whereas there was no significant change over time for 48 h pre-incubated HA samples (Figure 4). These results indicate that the low initial cell number observed after 0.5 h culture time was associated with a deficient number of proteins adhered to the HA surface, which would normally serve as focal adhesion points. Therefore, the pre-incubation process created a layer of proteins on the surface that is important to improve the early adhesion of cells (evaluated at 0.5 h). In contrast, at longer culture times (evaluated at 6 h), proteins present in the culture medium have sufficient time to adhere to the substrate as the culture progresses and therefore the number of cells adhered no longer reflects the pre-incubation step. A low degree of cell adhesion due to an insufficient amount of adhered protein was previously reported by Degasne et al. who used SEM to demonstrate that Saos-2 osteoblasts failed to adhere on the surfaces of titanium disks if a serum-free medium was used, whereas in the presence of FBS, greater cell colonization of the surface was observed.^28^ The importance of incubating HA with a protein-rich solution prior to cell seeding can be associated with the microstructure and the ionic modification of the medium caused by hydroxyapatite-based materials,^29,30^ which makes it a challenging biomaterial for cells to adhere to, although the reasoning behind the pre-incubation of these biomaterials is usually not made explicit.^31−33^ However, in the case of TCPS, an increase of cell number over time was only observed for non-pre-incubated samples (Figure 4), indicating that the modified surface chemistry of TCPS is already optimized for maximal cell adhesion.^34^ In fact, lower cell numbers were detected after 6 h of culture for pre-incubated TCPS compared to non-pre-incubated TCPS (Figure 4C). These results may be explained by the denaturalization of fibronectin on TCPS, which could cause aggregation of other proteins such as albumin.^35^ To conclude, the pre-incubation process has to be carefully optimized for each material, since as shown, incubating the samples with a medium containing 10% FBS results in a quicker and enhanced adhesion of cells to HA but does not have a positive effect for TCPS.

The osteoblast adhesion process was also modeled using a Langmuir-based model, which showed a good agreement with the experimental data (Figure 7). Moreover, as shown in Table 1, cell adhesion was directly linked to the prior incubation of the materials. For HA, the adhesion constant (*k*_a_^OB^) was higher after pre-incubation, which was linked to the presence of deposited protein on the substrate, as experimentally observed (Figure 2A) and modeled (Figure 3A) for BSA. For TCPS, the adhesion constant showed a similar correlation with pre-incubation time as with HA, while the cellular survival fraction displayed a downward trend with increasing pre-incubation time.

The good fit of the coupled cell-protein model (Figure 8) confirmed the accuracy of the transfer function between surface protein concentrations and subsequent cell adhesion. Interestingly, the main difference between the mathematical modeling of cell adhesion (Figure 7) and mathematical modeling with the protein-cell interplay (Figure 8) was observed for the 0 h pre-incubation period, when the samples that initially had no protein coverage consequently displayed poor initial cell adhesion. Similarly as was done in the current study, Chen et al. investigated the interplay of protein-cell adsorption and evaluated how this related to alumina’s particle size using a double-layer model based on the statistical Focker–Plank equation (protein-cell real-time coupling was neither included).^10^ The three key differences between the current study and the Chen et al. work are the following. First, Chen et al. assumed that protein and cell distributions over the surface were inhomogeneous, unlike in this work, where uniform distribution was considered. Second, the statistical Focker–Plank equation was used by Chen et al., whereas in the current work, a model based on Langmuir isotherm was proposed. Finally, the initial conditions of adhered cells in Chen et al. were determined from the amount of adsorbed protein, while in the present study it was assumed that no cells were adhered at the beginning of the experiment.^10^ These differences may explain why Chen et al. predicted an abrupt time evolution of adhered cell density (Figure 4 in Chen et al.), while in this work, cell adhesion increased continuously with time (Figure 8). Noteworthy in the current study, albumin was used as a protein model, while cell adhesion studies were performed with a cell culture medium supplemented with FBS. Despite this difference, as albumin is the most abundant of the almost 100 different types of proteins present in serum,^36^ the agreement is understandable.

The ability of the cells to spread on the surface after adhesion was also investigated experimentally (Figure 5) to gain more insight into the biomaterial-protein-cell interaction. For both HA and TCPS, pre-incubated samples consistently showed higher eccentricity values than samples that were not pre-incubated, this trend being particularly clear for HA (Figure 6). Pre-incubated samples contained a more populous distribution of adhered proteins on the surface as opposed to non-pre-incubated samples at the start of the culture period, therefore promoting initial cell spread. In addition, cell spread also appeared to increase over the culture period, regardless of pre-incubation conditions for both HA and TCPS samples, indicating that the spreading is not an instantaneous process. Overall, this confirmed that proteins play a key role in the mediation of cell spread, this being particularly important for biomimetic HA surfaces, which are challenging substrates.^30,31^ These results correlated well with a previous study where an increased spread of MC3T3-E1 pre-osteoblasts was found after 3 h of culture on HA pre-incubated with fibronectin and albumin as opposed to non-pre-incubated samples.^35^ In addition, Degasne et al. demonstrated that Saos-2 osteoblasts cultured in FBS displayed a flat and spread-out morphology, whereas cells grown in serum-free medium were round and globular in morphology.^28^

Despite the Langmuir isotherm’s proven suitability for capturing protein and cell dynamics, the method has several limitations worth highlighting. In regard to the protein portion of the model, BSA was assumed to have constant adsorption and desorption coefficients, which is a valid assumption only in the case of having physiological pH and temperature in the environment.^37^ Moreover, the kinetics of a single protein was contemplated, missing insight on the competitive processes that may occur between multiple proteins over time (i.e., Vroman effect). In regard to the cell portion, the model was designed for fully adherent cells (i.e., no detachment coefficient was used for cells). Furthermore, since the developed model does not consider cell proliferation, it is important to perform the culture study for a short time to best capture the cell adhesion dynamics. Finally, the model was not designed to capture cell-cell-dependent effects during the adhesion process. For this reason, a low cell seeding density (3500 cells/cm^2^) was used and future co-culture studies would require a more complex model. Interestingly, the experimental results of two materials with different chemical and physical properties correlated well with the model, each of them resulting in different model parameters (Table 1). The underlying reason is that the basic adhesion mechanism is similar and can be described using an exponential function. This indicates that the model has the potential to describe the interactions that proteins and cells have with a broad type of biomaterials.

In essence, the model can be considered a simplification of traditional *in vitro* cell cultures, which in turn are a convenient approximation of the *in vivo* environment. The physiological cellular environment is highly dynamic and interconnected, with cells able to sense and communicate with each other via a variety of cues and messenger molecules.^38^ In the current work, the cells were cultured under static conditions in standard well plates, although the environment created is known to poorly mimic the real physiological conditions.^2^ The protein and cellular adsorption behavior displayed in new and emergent approaches that may better reproduce the physiologic dynamic environment would be interesting to examine in the future.^29,39,40^

Overall, this study proves the importance that the pre-incubation step in a serum-supplemented medium may have on biomaterials, whereby it enhances early cellular adhesion responses. Moreover, it was shown that computational modeling, in this case through the usage of two Langmuir isotherm equations that allowed coupling of protein-cell adhesion, enabled an accurate prediction of cell adhesion behavior over time. As modeling is based on experimental data, more efficient development of new biomaterials could be achieved by compiling already available experimental raw data in open databases. This would permit researchers to get a basic idea of a biomaterial’s biological performance via computational modeling prior to experimental confirmation. Nonetheless, it is important to consider the practical limitations of the model and how well it fits the intended applications of the biomaterial study.

## 4 Conclusions

Mathematical computational models were successfully developed and applied to describe basic biological properties for both hydroxyapatite (HA) and tissue culture polystyrene (TCPS), using results obtained from experimental characterization. While almost half of the total amount of the model protein (bovine serum albumin, BSA) adsorbed on HA, only 18% of the previously adsorbed protein was released afterward. To account for the importance of the focal contact points offered by adhered proteins for the subsequent cell adhesion, the substrates were pre-incubated for different time periods before culturing cells. The total number and spread of adhered cells on the substrates was more pronounced on pre-incubated HA, whereas the pre-incubation step was not beneficial for TCPS. The Langmuir isotherm allowed accurate modeling of protein adsorption and desorption behavior, as well as the number of cells attached on the substrates. Furthermore, the same mathematical model could be used to couple the protein and cell adhesion interplay. Overall, we conveyed the potential use of mathematical and computational methods for biomaterial evaluation and parameter tuning in the context of *in vitro* cell culture, and the limitations of the model were discussed. In the future, the model could be the base of more sophisticated models that would include material and cell properties with the aim to obtain more refined predictions.

## 5 Materials and Methods

### 5.1 Fabrication of Biomimetic HA

Calcium-deficient hydroxyapatite (HA) was prepared by mixing a powder phase of α-tricalcium phosphate (α-TCP, Ca_3_(PO_4_)_2_) with 2.5% w/v sodium phosphate dibasic (Na_2_HPO_4_, #S7907, Merck) in water in a liquid-to-powder ratio of 0.65 mL/g. The α-TCP was prepared via mixing dicalcium phosphate anhydrous (CaHPO_4_, #40232.30, Alfa Aesar) and calcium carbonate (CaCO_3_, #10687192, Acros Organics) in a molar ratio of 2:1. The powder mixture was heated to 1450 °C in a furnace (Entech MF 4/16) on a zirconia plate setter for 5 h (total thermal treatment time of 18 h) and quenched in air. The resultant powder was dry-milled for 15 min at 300 rpm in a 500 mL zirconia mill jar within a planetary ball mill (PM400, Retsch), using 100 zirconia milling balls (⌀ = 10 mm diameter) for 100 g of powder.

The calcium phosphate cement was molded into circular Teflon molds (⌀ = 6 mm, h = 2 mm) and clamped against a glass slide with the aim to produce a flat top surface. The paste was set at 37 °C and 100% humidity for 4 h, after which the set disks were transferred to a 0.9% w/v sodium chloride solution, where the cements were further set for 10 days to ensure complete transformation into HA.

### 5.2 Physical Characterization of HA

HA disk samples were visualized by scanning electron microscopy (SEM). Prior to SEM analysis, samples were sputter-coated at 2 kV for 40 s with a thin layer of gold and palladium (Emitech SC7640, Quorum technologies). The coated samples were imaged in a field emission SEM (Zeiss LEO 1530, AB Carl Zeiss) at an acceleration voltage of 3 kV and a working distance of 9 mm, using an in-lens secondary electron detector.

The topographical features of the HA surface were scanned using an optical profilometer (Nexview NX2, ZYGO). A scan length of 65 μm was used and a 500 μm × 500 μm area of the material surface was scanned and stitched together using accompanying software (MX, ZYGO).

### 5.3 Biological Characterization of HA

#### 5.3.1 Protein Adsorption and Desorption

The adsorption and desorption of protein on HA were evaluated using albumin as a model protein. For the adsorption assay, a 10 mg/mL solution of bovine serum albumin (BSA, #A9418, Merck) in Dulbecco’s modified Eagle’s medium (DMEM, phenol-red free, #A1443001) was prepared, here referred to as BSA-medium. BSA medium (200 μL) was added to HA disks placed in a 96-well plate and aliquots were taken from independent triplicate sample sets at different intervals over a 72 h period. Afterward, the HA disks were transferred into well plates containing 200 μL BSA-free DMEM. Albumin desorption from the surface was then monitored via taking aliquots from independent triplicate sample sets at different time points over 72 h. Complete experiments were carried out twice.

The collected aliquots were analyzed using the bicinchoninic acid assay (BCA protein assay, ref no. 23225, Thermo Fisher Scientific) as per the manufacturer’s protocol. Concisely, the aliquots were diluted 10-fold in BSA-free DMEM and mixed in a 1:8 ratio with the working reagent in a 96-well plate. After 30 min of incubation at 37 °C, absorbance was measured at 562 nm using a microplate reader (Spark, TECAN). A standard curve of known albumin concentration samples was also prepared in a similar manner and used to transform absorbance values into total protein concentration.

#### 5.3.2 Cell Adhesion

MC3T3-E1 murine pre-osteoblasts (subclone 14, ATCC, Manassas, VA) were used. The cells were maintained in ascorbic-acid free Minimum Essential Medium Alpha (MEM-α, Gibco, #A1049001, Thermo Fisher Scientific) which was supplemented with 10% v/v fetal bovine serum (FBS, Thermo Fisher Scientific) and 1% v/v penicillin/streptomycin (Pen/Strep, #DE17-602E, BioWhittaker). The cells, which were grown in an incubator (Heracell 150, Heraeus) with a controlled humidified internal environment at 37 °C and 5% CO_2_, were split before reaching 80% confluence in a flask. The experiments were performed with MEM-α (Hyclone, #SH3026501, Cytiva) supplemented with 10% v/v FBS and 1% v/v Pen/Strep (referred as supplemented medium).

HA disks were sterilized by immersion in 70% ethanol for 2 h and were subsequently rinsed abundantly with autoclaved distilled water and dried in air. Prior to cell seeding, HA disks (placed in 96-well plates) and tissue culture polystyrene (TCPS) of a 96-well plate format were pre-incubated for 24 or 48 h in 200 μL of supplemented medium. Non-pre-incubated samples are indicated as “0 h.”

Before seeding, MC3T3-E1 cells in suspension in a serum-free MEM (#51200046, Thermo Fisher Scientific) were stained with CellTracker Green CMFDA dye (1 μM, #C2925, Thermo Fisher Scientific) for 20 min. Afterward, the cells were seeded on HA disks and TCPS (3500 cells/cm^2^), which had been pre-incubated with supplemented medium for 0, 24, or 48 h. The samples were imaged at 0.5, 1, 2, 4, and 6 h after seeding with a fluorescent microscope (IX73 Inverted Microscope, Olympus), using independent samples. Triplicate samples were included and each sample was imaged five times at different locations.

Image analysis was performed using CellProfiler software (version 3.1.5).^41^ Cells were segmented and cell counts were obtained through quantification of all segmented cells per image. The eccentricity of segmented cells was evaluated to characterize their shape, where the eccentricity of cells was measured by the ratio of the distance between the ellipsoid foci and the ellipsoid major axis length.^42^ A value close to 1 indicates the shape of an elongated ellipse, and herein that a cell is spread. For both cell count and eccentricity, an average was taken for all images for each sample per time point.

### 5.4 Statistics of Experimental Characterization

All data points were plotted as the mean ± standard deviation of the replicates for all experiments performed. Protein samples were compared using a one-way two-sided ANOVA, while cell count and eccentricity samples were compared using a two-sided ANOVA General Linear Model, both at a significance level of 0.05. Post-hoc Tukey testing was performed to inspect pair-wise significant differences between different sample groups and Dunnett’s testing was performed to investigate significant differences between a control value and the test sample groups. All significance testing was performed using Minitab 17.

### 5.5 Modeling of Protein Adsorption and Desorption on HA

**Figure 9:**
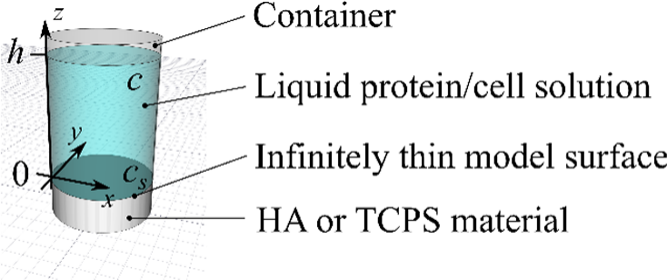
Experimental sketch of the incubated material, along with the introduced variables for BSA solution concentration c and BSA surface concentration *c*_s_.

To model protein adsorption and desorption, the coordinate system shown in Figure 9 was introduced. To achieve ease of use, a simple mathematical model suitable to represent the experimental physical conditions was designed. The model was based on three key assumptions:

A1. The liquid solution does not move (i.e., the velocity field is zero) and therefore the transport of proteins occurs only through the diffusion process.
A2. The protein concentration is homogeneous and constant through the cylindrical cross section of the cylindrical container. The concentration varies only as a function of height above the HA disk.
A3. The HA disk is modeled as infinitely thin and thus any transport inside the material is neglected.

The diffusion of BSA in a fluid phase has been studied by Gaigalas et al., who characterized the diffusion coefficient D with respect to temperature, solution pH, and protein concentration.^37^ Within the current experimental study, the temperature of the solution was maintained at 37 °C, BSA concentration in the fluid phase varied between 0 and 10 mg/mL and a bicarbonate buffering environment was present in the solution to buffer pH changes. Based on experimental parameters, the BSA diffusion coefficient was set to *D* ∼ 0.3 mm^2^/h ∼ 9 × 10^–7^ cm^2^/s and it was assumed to remain approximately constant over time.

The two key variables in the system are fluid phase protein concentration (*c*, μg/mL) and surface protein concentration (*c*_s_, μg/mm^2^) (Figure 9). Assumption A2 renders the fluid phase concentration *c* a function of the z coordinate only, and the surface concentration *c*_s_, a scalar value. The time evolution of the fluid phase protein concentration is governed by a diffusion equation (eq 1)
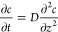
1

The equation requires an initial condition as well as boundary conditions at the top (*z* = *h*) and at the bottom (*z* = 0) of the domain. An initial BSA concentration in the fluid phase of 9500 μg/mL (BSA-rich medium concentration, Figure 2A) was used. It was assumed that at the beginning of the experiment and during sampling, the measurement process led to mixing and the formation of a uniform protein concentration in the solution. At the top, zero-flux condition (∂*c*/∂*z* = 0) was applied since proteins cannot escape through the upper boundary of the fluid solution. The boundary condition at the bottom (in contact with HA or TCPS) was determined by the Langmuir isotherm as
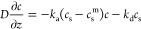
2where *k*_a_ is the adsorption coefficient, *k*_d_ is the desorption coefficient, and *c*_s_^m^ is the maximum surface protein concentration. It was assumed that surface protein concentration is always smaller than the maximum, that is, *c*_s_ < *c*_s_^m^. Finally, eq 3 states that protein adsorption to the surface leads to changes in the surface protein concentration over time, i.e.,
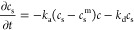
3

Equation 3 denotes the Langmuir isotherm as used in surfactant modeling.^24^ It is used in flux form, rewritten for protein concentration. The first term on the right-hand side of the equation is positive (*c*_s_ < *c*_s_^m^) and models protein attachment on the surface. The second term is negative and models protein detachment from the surface. The protein adsorption and desorption experiments were constructed to focus on each term individually. For the adsorption experiment, initially *c*_s_ = 0, which thus allows the first term to be tested. For the desorption experiment, initially *c* = 0, which thus allows the second term to be tested. When the system reaches the steady state, there is no change in time, i.e., . The protein concentrations in fluid phase and on the surface reach their final values, *c*^f^ and *c*_s_^f^, respectively. For given *k*_a_ and *k*_d_ values, the maximum surface protein concentration can then be obtained from eq 4 as
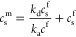
4where *c*^f^ and *c*_s_^f^ are final steady-state fluid phase and surface BSA concentrations, respectively. The *c*^f^ and *c*_s_^f^ values were obtained from adsorption experiments. After determining *c*_s_^m^ from eq 4, we solved eqs 1–3 numerically as described in the Supporting Information.

The remaining adsorption coefficient (*k*_a_) and desorption coefficient (*k*_d_) parameters were fitted in the following manner; *k*_a_ was sampled in the interval *k*_a_ ∈ (2.5–12.5) × 10^–4^ mL/(μg·h) with steps of 0.53 × 10^–4^ mL/(μg·h), and *k*_d_ was sampled in the interval *k*_d_ ∈ (0.01–0.20) h^–1^ with steps of 0.01 h^–1^. For each combination of adsorption and desorption coefficient, *c*_s_^m^ was estimated using eq 4. Then, two simulations corresponding to both protein adsorption and desorption experiments were carried out. For each simulation, the predicted average protein concentration in the solution was extracted over time and compared with the experimental measurement. The error between the experimental results and the modeling results was computed using the formula
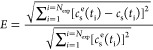
5where *N*_exp_ is the number of experimental measurements, *c*_s_^e^ (*t*_i_) is the experimental result, and *c*_s_ (*t*_i_) is the model prediction, both at time *t*_i_. The errors from the two simulations with the same *k*_a_ and *k*_d_ coefficients were added and the parameter combination providing the smallest error was identified, thus providing fitted values for *k*_a_ and *k*_d_ coefficients.

### 5.6 Modeling of Cell Adhesion

The mathematical model for cell adhesion is based on the same three assumptions (A1–A3) explained in Section 5.5, as well as the following:

A4. The cell settling time (i.e., time over which cells deposit on the surface and form a uniform layer) is short in comparison to the experimental duration, and therefore this process is neglected.
A5. A fraction of cells fail to remain viable during seeding and do not take part in the adhesion process.
A6. Attached living cells do not detach during the course of the experiment.

The cell adhesion model proposed is similar to the model for protein adsorption/desorption. In particular, the model for cell adhesion simply states that the cells are either part of a “free” cell layer or of a layer of cells attached to the substrate. Therefore, the two variables in the system are “free” cell concentration (*c*^OB^, cells/cm^2^) near the substrate and attached cell concentration (*c*_a_^OB^, cells/cm^2^). The cell adhesion model constitutes a simple two-variable model, which describes the time evolution of both free and attached cell concentrations. The free cell concentration over time can be written as
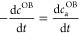
6

Equation 6 postulates that cells leaving the free layer end up in the attached layer. The cell adhesion was modeled through an equation similar to the one used for the surface concentration of proteins (eq 3) but with the desorption term neglected
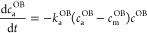
7

Here, *k*_a_^OB^ is the cell adhesion coefficient (cm^2^/cells·h) and *c*_m_^OB^ is the maximum concentration of cells on the surface for a given growth area (cells/cm^2^). The maximum concentration *c*_m_^OB^ is estimated based on the typical size of a cell. It was assumed that each spread cell occupies a square region with side length of 25 μm, which therefore implies that *c*_m_^OB^ = 1.6 ×10^5^ cells/cm^2^. The adhesion coefficient *k*_a_^OB^ is left as free fitting function. The initial concentrations of the cell adhesion modeling were
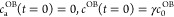
8where *c*_0_^OB^ is the seeded density of cells and γ is the fraction of the cells that reach the HA surface in a viable state. The fraction γ was obtained by taking the mean of the last two experimental cell counts (at 4 and 6 h, Figure 7) and dividing the result by the seeded cell count. The set of ordinary differential equations with the initial conditions (eqs 6–8) were solved analytically. The attached cell concentration evolution in time was expressed as
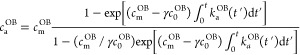
9

Equation 9 captured the expected result for a large period of time, whereby *c*_a_^OB^ = γ*c*_0_^OB^. That is, after a sufficiently long period, all viable cells will be attached. The unknown function *k*_a_^OB^ (*t*) was treated in two different ways. First, for the initial estimate, it was assumed that *k*_a_^OB^ does not vary over time. The integral in time was simplified to ∫_0_^*t*^*k*_a_^OB^ (*t*′)d*t*′ = *k*_a_^OB^*t*. A single *k*_a_^OB^ constant was found for each cell adhesion experiment by fitting a value that yields the smallest error (eq 5, redefined using cell concentration). The error bound for *k*_a_^OB^ was found by identifying the interval, in which the error (eq 9) would not increase by more than 50%. Subsequently, the modeling of cell adhesion was coupled to that of protein adsorption. The cellular dependence on surface protein for adhesion was accounted for as
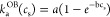
10To facilitate the modeling process, it was postulated that for zero protein concentration, the cell adhesion coefficient should be zero, as in *k*_a_^OB^ (0) = 0. The constants *a* and *b* were adjusted to match the cell adhesion experiments with the coupled model. This function was compared with the constant *k*_a_^OB^ values obtained for certain averaged protein surface concentrations. Finally, the integral ∫_0_^*t*^*k*_a_^OB^ (*t*′)d*t*′ was evaluated at each time instant *t* for all cell adhesion experiments based on the modeled surface protein concentration.

## Supporting Information Available

Characterization of HA through X-ray diffraction analysis (Figure S.I. 1); numerical solution of the protein/cell adhesion models (eqs S1–S4); cell morphology at higher magnifications (Figures S.I. 2 and S.I. 3); and visualization of relevant protein concentrations for coupling to cell adhesion model (Figure S.I. 4) (PDF)
