# Supplementary material for: Experimental Characterization and Mathematical Modeling of the Adsorption of Proteins and Cells on Biomimetic Hydroxyapatite
Source: ACS Omega. 2021 Dec 22;7(1):908–20. doi: 10.1021/acsomega.1c05540 (PMC8757448; doi:10.1021/acsomega.1c05540)
Supplement: Supplementary file 1 — ao1c05540_si_001.pdf [file ao1c05540_si_001.pdf]

Supporting Information

Experimental characterisation and mathematical modelling of the adsorption of proteins and cells on biomimetic hydroxyapatite

Abdul-Raouf Atif[1], Uģis Lācis[2], Håkan Engqvist[1], Maria Tenje[1,3], Shervin Bagheri[2], Gemma Mestres[1,3*]

[1] Dept. of Materials Science and Engineering, Uppsala University, 751 22 Uppsala, Sweden

[2] Dept. of Engineering Mechanics, FLOW Centre, KTH Royal Institute of Technology, 114 28 Stockholm, Sweden

[3] Science for Life Laboratory, Uppsala University, 751 22 Uppsala, Sweden

* Corresponding author: Gemma Mestres

Postal Address: Department of Materials Science and Engineering

Uppsala University, Box 35, 751 03 Uppsala, Sweden

Telephone: +46 18 471 3235

E-mail: gemma.mestres@angstrom.uu.se

Data included

1) Characterization of HA through X-ray diffraction analysis (Fig. S.I. 1); 2) numerical solution of the protein/cell adhesion models (equations S1-S4); 3) cell morphology at higher magnifications (Fig. S.I. 2, 3); and 4) visualization of relevant protein concentrations for coupling to cell adhesion model (Fig. S.I. 4).

1. XRD of α -TCP Powder and Hydroxyapatite

The crystalline phases of the powder phase and the HA set for 10 days in 0.9% $NaCl_{(aq)}$ were evaluated by X-ray diffraction analysis (XRD), with a Bruker D8 Advanced (Bruker Daltonics) using Cu Kα radiation (λ = 1.5418 Å). A scanning range of 3 to 60°, with a step size of 0.03° per step, and a dwell time of 0.2 seconds was performed. For phase identification, the diffraction patterns were compared with the Joint Committee on Powder Diffraction Standards for α-TCP (JCPDS #09-0348) and HA (JCPDS #82-1943). The XRD spectra of the α-TCP powder and the one of the calcium phosphate cement set for 10 days, which confirmed a complete transformation into hydroxyapatite, are shown in Figure S.I. 1.

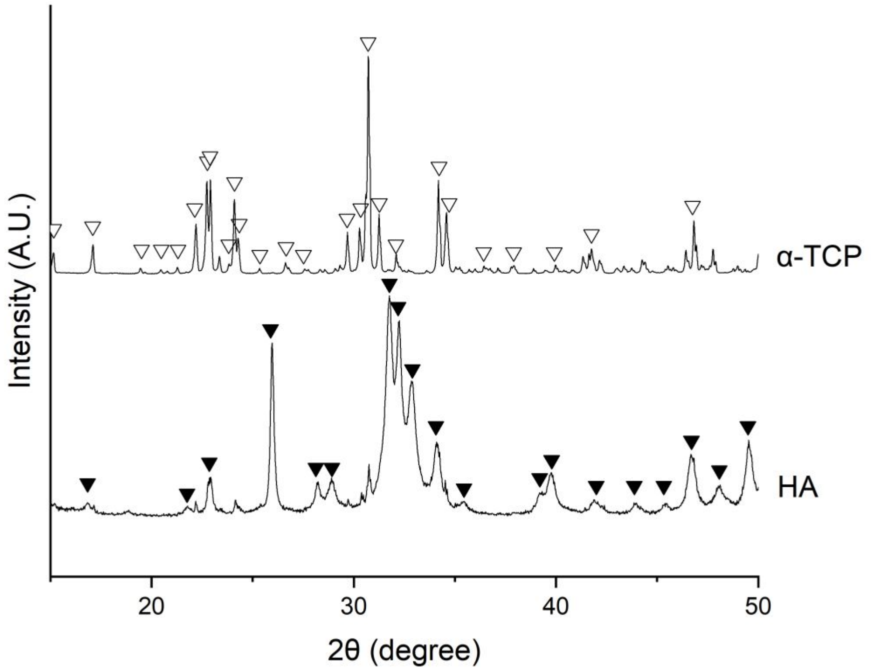

Figure S.I. 1. XRD trace of α-TCP and HA set for 10 days in PBS in an $NaCl_{(aq)}$ solution. White and black triangles point to the Powder Diffraction Standards for α-TCP and HA, respectively.

2. Numerical solution of diffusion equations

Equations 1-3 in the main paper were discretised in time and were solved numerically using a finite element method (FEM) with FreeFEM solver (version 4.6). Implicit-Euler time stepping was used for the diffusion equation (S1) and semi-implicit time stepping scheme was used for surface adsorption (S3):

$$\frac{c^{n+1} - c^{n}}{\Delta t} = D\frac{\partial^2 c^{n+1}}{\partial z^2}, \tag{S1}$$

$$D\frac{\partial c^{n+1}}{\partial z} = -\,k_a\left(c_s^{n+1} - c_s^{m}\right)c^{n} - k_d c_s^{n+1}, \tag{S2}$$

$$\frac{c_s^{n+1} - c_s^{n}}{\Delta t} = -\,k_a\left(c_s^{n+1} - c_s^{m}\right)c^{n} - k_d c_s^{n+1}, \tag{S3}$$

where Δt was the discrete time step, *n* was the current time index and *n*+1 was the next time index. The surface concentration equation (S3) was rearranged to obtain

$$c_s^{n+1} = \frac{c_s^{n} + k_a \Delta t c_s^{m} c^{n}}{1 + k_a \Delta t c^{n} + k_d \Delta t}. \tag{S4}$$

This result was inserted in boundary condition (S2) to reduce number of equations to be solved. The variables at $t = 0$ ($n = 0$) were initiated according to initial conditions and solution was integrated forward in time with time step $\Delta t = 0.001$ h. It was checked that reducing the time step and FEM mesh resolution did not change the results significantly.

3. Morphology of cells adhered on HA and TCPS

MC3T3-E1 cells were stained with CellTracker green CMFDA dye (1 μM) and cultured on HA and TCPS for up to 6 hours. Cells were imaged at different magnifications; the lower magnification is shown in the manuscript (Figure 5) and higher magnifications are shown below. (Figure S.I. 3 and S.I. 4).

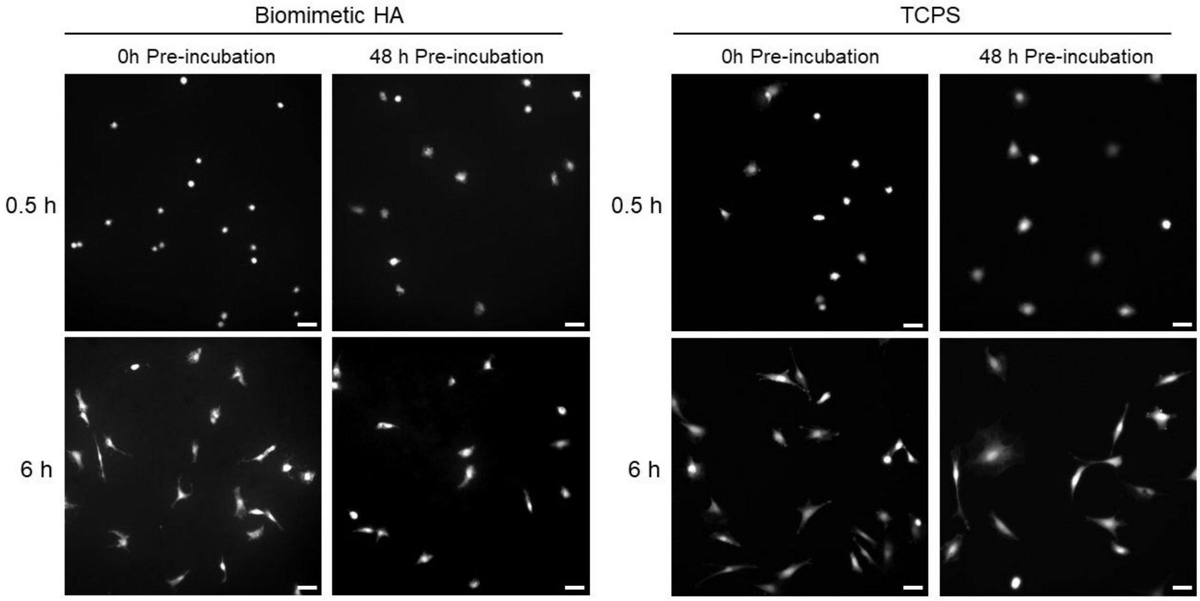

Figure S.I. 2. Cells adhered on HA and TCPS after 0.5 and 6 h of culture, with each sample pre-incubated for 0 or 48 h (scale bar = 50 μm).

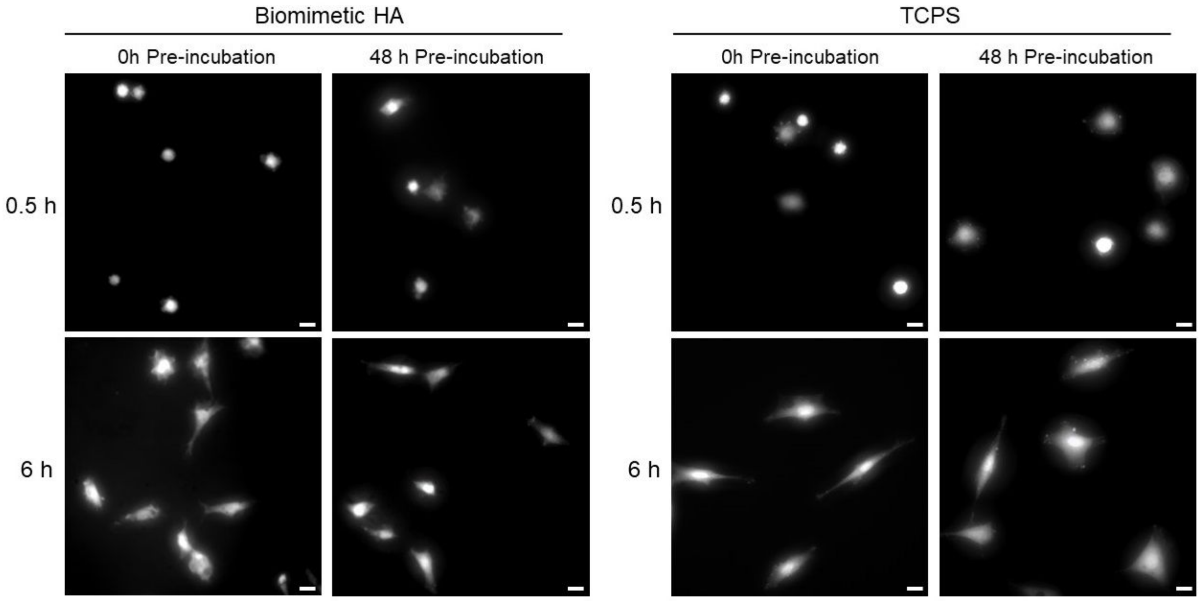

Figure S.I. 3. Cells adhered on HA and TCPS after 0.5 and 6 h of culture, with each sample pre-incubated for 0 or 48 h (scale bar = 20 μm).

4. Coupling of Protein adsorption and Cell adhesion models

The protein adsorption and cell adhesion models were coupled using the average protein concentration indicated by the bounds in Figure S.I. 4.

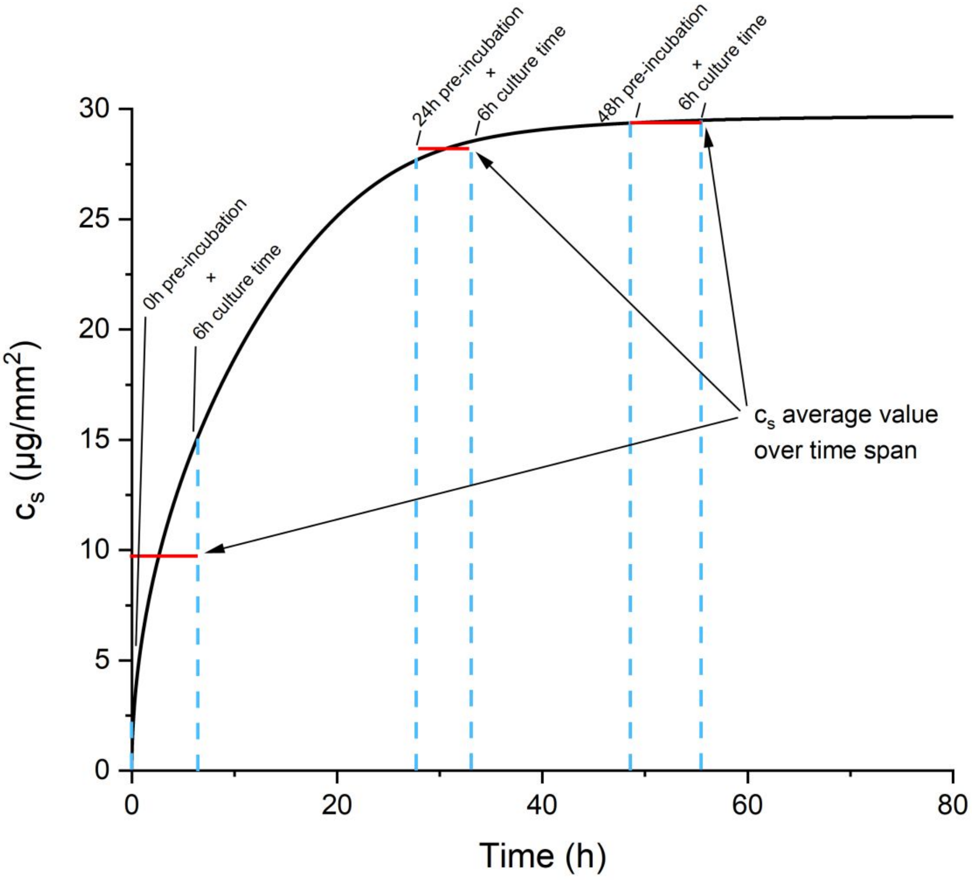

Figure S.I. 4. Surface BSA protein concentration predicted by a numerical model (Figure 3A), overlaid with the corresponding cell adhesion experiment time span.
